# Direct and Transcutaneous Vagus Nerve Stimulation for Treatment of Tinnitus: A Scoping Review

**DOI:** 10.3389/fnins.2021.680590

**Published:** 2021-05-28

**Authors:** Natalia Yakunina, Eui-Cheol Nam

**Affiliations:** ^1^Institute of Medical Science, School of Medicine, Kangwon National University, Chuncheon, South Korea; ^2^Department of Otolaryngology, School of Medicine, Kangwon National University, Chuncheon, South Korea

**Keywords:** tinnitus, vagus nerve stimulation, transcutaneous vagus nerve stimulation, neuromodulation, auricular branch of vagus nerve

## Abstract

Recent animal research has shown that vagus nerve stimulation (VNS) paired with sound stimuli can induce neural plasticity in the auditory cortex in a controlled manner. VNS paired with tones excluding the tinnitus frequency eliminated physiological and behavioral characteristics of tinnitus in noise-exposed rats. Several clinical trials followed and explored the effectiveness of VNS paired with sound stimuli for alleviating tinnitus in human subjects. Transcutaneous VNS (tVNS) has received increasing attention as a non-invasive alternative approach to tinnitus treatment. Several studies have also explored tVNS alone (not paired with sound stimuli) as a potential therapy for tinnitus. In this review, we discuss existing knowledge about direct and tVNS in terms of applicability, safety, and effectiveness in diminishing tinnitus symptoms in human subjects. This review includes all existing clinical and neuroimaging studies of tVNS alone or paired with acoustic stimulation in tinnitus patients and outlines the present limitations that must be overcome to maximize the potential of (t)VNS as a therapy for tinnitus.

## Introduction

Tinnitus is the perception of a phantom auditory sensation in the absence of an external sound source. It is one of the most prevalent auditory disorders, affecting 10–15% of the population, sometimes severely impairing quality of life (Davis and Rafaie, 2000; Baguley et al., 2013). In this paper we will discuss only subjective idiopathic tinnitus. The psychological model of tinnitus suggests that the overall annoyance of the tinnitus is a result of the tinnitus characteristics and the psychological make-up of each individual patient (Tyler et al., 1992). Treatments can be focused on reducing the tinnitus sensation (e.g., pharmacological) or on reducing the reactions to the tinnitus (e.g., psychological and cognitive training). A variety of treatments exist, including acoustic stimulation-based and brain stimulation therapies, but most tinnitus cases remain refractory to treatment.

One of the existing tinnitus models describes the tinnitus-generation mechanism as “maladaptive plastic re-organization of the auditory cortex” and suggests that tinnitus may develop as a result of auditory deafferentation related to peripheral hearing loss (Muhlnickel et al., 1998; Norena and Eggermont, 2003; Shore et al., 2016). Reduced output from the affected cochlear region could induce loss of lateral inhibition from the damaged frequency areas, which may lead to elevated neural synchrony and hyperexcitability of the central auditory system (Eggermont and Roberts, 2004).

Vagus nerve stimulation (VNS) via surgical implantation is an FDA-approved procedure for the treatment of epilepsy and depression that is believed to trigger the release of neuromodulators in the brain (Schachter and Saper, 1998; Groves and Brown, 2005; Milby et al., 2008). Presentation of a tone together with a neuromodulator release, i.e., targeted neuromodulation, could increase the proportion of auditory cortical neurons that respond to the paired tone (Bakin and Weinberger, 1996; Kilgard and Merzenich, 1998; Martins and Froemke, 2015). In 2011, Engineer et al. (2011) reversed tinnitus-related cortical plastic maladaptation in a targeted and controlled way by applying VNS combined with acoustic stimulation. They demonstrated that VNS paired with interleaved multiple tones spanning the hearing range but excluding the tinnitus frequency eliminated the behavioral and neural manifestations of tinnitus in noise-exposed rats (Engineer et al., 2011). A few human studies that followed suggested that VNS paired with tones stripped of the tinnitus frequency might improve tinnitus-related symptoms (De Ridder et al., 2014a, 2015; Tyler et al., 2017).

Transcutaneous VNS (tVNS), applied at either the auricular or cervical branch of the vagus nerve, has been adopted as a non-invasive alternative to VNS (Yap et al., 2020). Multiple neuroimaging studies have confirmed that tVNS activates the same brain networks and pathways as those by direct VNS (Kraus et al., 2007, 2013; Frangos et al., 2015; Yakunina et al., 2017). A few clinical trials have explored the safety of tVNS alone (Kreuzer et al., 2012, 2014; Ylikoski et al., 2017, 2020), and neuroimaging studies have mapped its neuromodulatory effects (Lehtimaki et al., 2013; Hyvarinen et al., 2015; Yakunina et al., 2018) on the brain. The effectiveness of tVNS combined with acoustic stimulation has been explored by only two clinical trials to date (Lehtimaki et al., 2013; Shim et al., 2015).

Tailor-made notched music training (TMNMT) has been developed to reverse maladaptive plastic changes in the auditory cortex by enhancing lateral inhibition (Pantev et al., 2004). TMNMT employs music with an octave (or half octave) band centered on the patient’s tinnitus, with the tinnitus frequency filtered out (notched) to augment lateral inhibition to the notched region (Okamoto et al., 2010; Pantev et al., 2012). Thus, TMNMT could be applied instead of the multiple tones stripped of the tinnitus frequency (paired with VNS) used in the 2011 study by Engineer et al. (2011) to increase the ratio of non-tinnitus frequency neurons and suppress cortical overrepresentation of the tinnitus frequency.

A recent systematic review reviewed nine studies on (t)VNS effect in reducing tinnitus symptoms (Stegeman et al., 2021). The review concluded that due to methodological limitations and low reporting quality of the studies, the effect of VNS on tinnitus remains unclear. In this review, in addition to discussing the applicability, safety, and effectiveness of direct and tVNS for treating tinnitus, we also review general knowledge about the mechanism, stimulation parameters, anatomical basis, and electrode placement in tVNS as well as relevant neuroimaging experiments. We then consider in detail how tVNS is currently applied for tinnitus treatment, all existing clinical and neuroimaging studies on tVNS alone or paired with sound stimuli in tinnitus patients, and its effectiveness. We discuss the quality of the existing theoretical basis for VNS for tinnitus. Finally, we outline fundamental gaps that must be overcome to maximize the efficacy of tVNS as a part of tinnitus therapy and discuss possible future directions for facilitating tNVS therapy.

## VNS: Targeting Plasticity

The cervical branches of the vagus nerve are mainly afferent sensory fibers that synapse in the nucleus tractus solitarius (NTS) and then project to the noradrenergic locus coeruleus (LC) in the brainstem and the cholinergic nucleus basalis (Leslie et al., 1982; Berthoud and Neuhuber, 2000) in the basal forebrain. They are involved in the release of neuromodulators such as acetylcholine, norepinephrine, serotonin, and brain-derived neurotrophic factor (Detari et al., 1983; Hassert et al., 2004; Follesa et al., 2007; Manta et al., 2009; Raedt et al., 2011) and subsequently influence the limbic, reticular, and autonomic centers of the brain (Sumal et al., 1983; Berntson et al., 1998; Berthoud and Neuhuber, 2000; Henry, 2002; Lulic et al., 2009; Manta et al., 2009). These neuromodulators seem to play key roles in promoting plastic changes (Bear and Singer, 1986; Kirkwood et al., 1999; Bramham and Messaoudi, 2005; Seol et al., 2007) (see Hays et al., 2013 for a review), although the precise mechanism of VNS neuromodulation remains unknown.

VNS paired with sensory stimuli or an active task has been shown to drive reorganization in various parts of the cerebral cortex. Pairing VNS with movement reorganizes the motor cortex (Porter et al., 2012), and VNS paired with physical rehabilitation improves recovery of motor function after a stroke (Khodaparast et al., 2013; Dawson et al., 2016; Pruitt et al., 2016; Kimberley et al., 2018). VNS paired with sensory stimuli restores sensory function after neurological injury (Meyers et al., 2019; Darrow et al., 2020a,b). Pairing extinction training with VNS reduces conditioned fear by modulating plasticity in the pathway from the ventromedial prefrontal cortex to the amygdala (Peña et al., 2014; Childs et al., 2015; Noble et al., 2019).

VNS paired with tones could induce reorganization of the tonotopic map in the auditory cortex, enhancing cortical responses to the paired tone and altering the fields across the entire auditory pathway, increasing the percentage of each field that responds to the paired tone frequency (Engineer et al., 2011, 2015, 2017; Shetake et al., 2012; Buell et al., 2019; Adcock et al., 2020).

## tVNS: A Non-Invasive Alternative

### Anatomical Basis for tVNS

tVNS can be performed on the cervical branch of the vagus nerve in the neck. Cervical tVNS has been used to treat various disorders such as migraines, cluster headaches, and asthma (Yuan and Silberstein, 2016; Frangos and Komisaruk, 2017). In tinnitus patients, tVNS has been exclusively applied in the ear on the auricular branch of the vagus nerve (ABVN); hence, tVNS refers to auricular tVNS in this paper.

**FIGURE 1:**
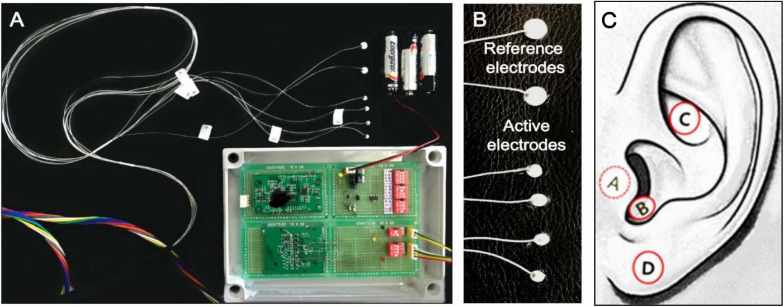
**(A)** Acustom-built MRI-compatible tVNS stimulator used in the tVNS fMRI studies by Yakunina et al., 2017, 2018 **(B)** Six silver electrodes (four active, two reference electrodes). **(C)** tVNS stimulation locations: inner surface of the tragus (A), inferoposterior wall of the external acoustic meatus (B), cymba conchae (C), and earlobe (sham; D).

tVNS is usually performed in the left ear to avoid cardiac complications, as efferent vagal fibers to the heart are predominantly located on the right side (Nemeroff et al., 2006; Ogbonnaya and Kaliaperumal, 2013). The ABVN originates from the superior ganglion of the vagus nerve and innervates the external acoustic meatus and auricle (Tekdemir et al., 1998; Kiyokawa et al., 2014). A widely cited cadaver study on the AVBN conducted by Peuker and Filler (2002) concluded that the ABVN innervates most prominently the antihelix, tragus, and cymba conchae, but other studies have suggested denser innervation on the posterior wall of the external acoustic meatus (Kiyokawa et al., 2014; Watanabe et al., 2016). However, a number of functional magnetic resonance (fMRI) studies have shown a practical preference for the inner tragus (Dietrich et al., 2008; Kraus et al., 2013; Yakunina et al., 2017; Badran et al., 2018) and cymba concha (Frangos et al., 2015; Yakunina et al., 2017; Wang et al., 2018) over the ear canal’s posterior wall as sites for stimulation of the ABVN. Figure 1 demonstrates the tVNS locations used in a previous fMRI study (Yakunina et al., 2017). A recent extensive review concluded that the current literature lacks a clear consensus on the location most densely innervated by the ABVN, but given the existing research, it is reasonable to assume that the cymba concha and inner tragus are suitable locations for vagal modulation (Butt et al., 2020). Between the two locations, tVNS was suggested to activate the vagal pathway slightly more effectively at the concha than at the tragus (Yakunina et al., 2017). Locating and fixing an electrode might be structurally easier in the tragus (using a clip-on or insert-type electrode) than in the concha. However, a fMRI study showed that the maximal tolerable intensity of electrical stimulation was significantly higher in the conchae than in the tragus, so the degree of brain (de)activation was stronger with concha stimulation (Yakunina et al., 2018).

### Optimal tVNS Parameters

A variety of pulse frequencies, widths, and intensities and shapes of electrical currents have been evaluated (see Yap et al., 2020 for a review), but no consensus on the optimal values has been achieved. In fMRI studies of normal human subjects using a pulse width of 250 μs and stimulation frequency of 25 Hz, tragus stimulation activated the left LC (Dietrich et al., 2008), and concha stimulation activated the LC and NTS (Frangos et al., 2015; Table 1). Using 500 μs at 25 Hz, stimulation of both locations activated the LC and NTS (Yakunina et al., 2017). In a subsequent study of tinnitus patients using the same tVNS parameters (25 Hz and 500 μs), Yakunina et al. (2018) replicated fMRI activation of the NTS and LC. However, a later study using the same parameters demonstrated similar cortical effects but not activation of the brainstem nuclei (Badran et al., 2018). That study set the stimulation intensity as 200% of the sensory threshold, whereas the Frangos et al. (2015) and Yakunina et al. (2017) used a current intensity just a step below the pain threshold (Table 1). Therefore, based on neuroimaging experiences so far, the following tVNS parameters could be applied to activate the classical vagal pathway: stimulation frequency of 25 Hz, pulse width of 250 or 500 μs, and tolerable maximal intensity.

**TABLE 1:** Parameters used in the tVNS fMRI studies.

Study	Subjects	Location	Pulse width (μs)	Intensity level	LC/NTS activation
Dietrich et al. (2008)	Normal	Tragus	250	4–8 mA	Left LC
Frangos et al. (2015)	Normal	Concha	250	Just below pain threshold. 0.3–0.8 mA 0.43 ± 0.14	LC, NTS
Yakunina et al. (2017)	Normal	Tragus, concha	500	Just below pain threshold. 0.2–1.8 mA 0.77 ± 60.42 tragus 0.91 ± 60.47 concha	LC, NTS
Yakunina et al. (2018)	Tinnitus	Tragus, concha	500	Just below pain threshold. 0.1–1.8 mA 0.71 ± 0.43 tragus 0.80 ± 0.47 concha	LC, NTS
Badran et al. (2018)	Normal	Tragus	500	200% of sensory threshold. Sensory threshold: 1.57 ± 0.48 mA	None

*All studies used a stimulation frequency of 25 Hz.*

To our knowledge, only one non-tinnitus study paired tVNS with sensory stimuli (Llanos et al., 2020). tVNS paired with speech stimuli robustly enhanced speech category learning and retention of correct stimulus-response associations. The authors used pulse width of 150 μs, frequency of 25 Hz, and amplitudes below each participant’s perceptual level (1.24–1.67 mA). However, the observed behavioral changes immediately followed the single 25-min tVNS session and thus did not seem to be due to long-term plastic changes in sensory representation of the stimuli, but most likely resulted from processes related to the adjustment of the functional mapping between representations of stimulus signals and examined stimuli categories (Llanos et al., 2020). Polak et al. (2009) measured vagus somatosensory evoked potentials (VSEP) in healthy participants as a measure of vagus brainstem nuclei activity, and concluded that 8 mA without pain perception was the optimal tVNS intensity to maximize VSEP. However, it should be considered that the amount of stimulation actually delivered to the tissue depends on the electrode material and tissue impedance (and thus electrode location), and therefore stating the exact amplitude as optimal may not be proper (Yap et al., 2020). Furthermore, although individual tolerances may widely vary, 8 mA still appears well above pain threshold of most people (Table 1). Polak et al. (2009) also acknowledged that VSEP amplitudes are directly correlated to stimulation intensity, and thus our previous conclusion that a maximal intensity without feeling of pain may be optimal for tVNS appears valid. Altogether, further studies that would pair tVNS with sensory stimuli are needed to establish more optimized parameters for improving the efficiency of the tVNS method.

It is sensible to consider how tVNS compares to VNS in terms of its effectiveness for other disorders. A recent systematic review of tVNS in epilepsy reported the overall mean seizure reduction of approximately 42% in the treatment group, with about 43.4% of patients being responders, which was similar to the results of direct VNS with 50.6% of responders and mean seizure reduction of 44.6% (Wu et al., 2020). In depression, several systematic reviews concluded that the existing evidence for VNS efficacy in depression is not of sufficient quality to make clear conclusions (Lv et al., 2019). Non-randomized and not controlled studies report 42–53% response rate to VNS in depression (Nahas et al., 2005; Bajbouj et al., 2010), while a tVNS study reported similar response rate of 27–80% depending on the treatment duration (Rong et al., 2016). Therefore, tVNS appears to be similar to VNS in terms of its effectiveness for other disorders, and both treatments have about 50% rate of success.

## Neuroimaging Studies: VNS and tVNS Effects on Brain Activity

The effects of VNS have been studied using various neuroimaging methods such as single-photon emission computed tomography (SPECT), positron emission tomography (PET), and fMRI (Chae et al., 2003). Results using all methods suggested that VNS induces immediate as well as lasting changes in the thalamus, cerebellum, orbitofrontal cortex, limbic system, hypothalamus, and medulla (Bohning et al., 2001; Lomarev et al., 2002; Narayanan et al., 2002; Chae et al., 2003). It is noteworthy that the majority of the structures VNS affects are subcortical, while the paired VNS approach targets sensory cortex by pairing VNS-triggered release of neuromodulators with a sensory stimulus.

One of the most consistent findings in studies of acute VNS effects is diminished activity and reduced cerebral blood flow in the limbic system, namely the amygdala, hippocampus, cingulate cortex, ventral anterior cingulum, and parahippocampal gyrus (Zobel et al., 2005; Vonck et al., 2008). There is no universally accepted list of structures that comprise the limbic system, but the above areas are believed to constitute its core (Mega et al., 1997; Rajmohan and Mohandas, 2007).

**FIGURE 2:**
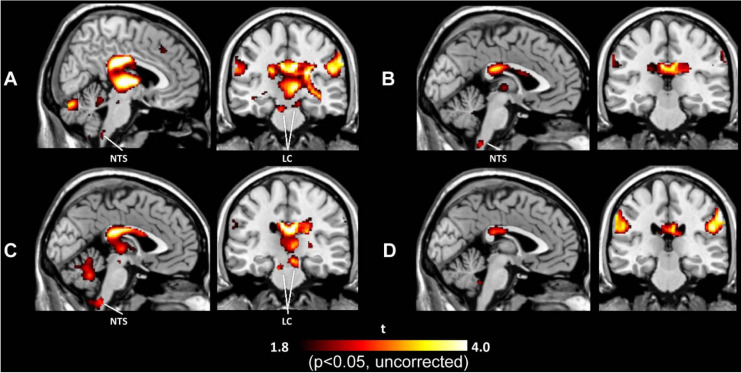
Activation maps induced by tVNS in the tragus **(A)**, inferoposterior wall of the external acoustic meatus **(B)**, concha **(C)**, and earlobe **(D)**. tVNS at the tragus and concha activated the locus coeruleus (LC) and nucleus of the solitary tract (NTS), while tVNS at the ear canal and earlobe did not activate either brain center.

Brain activation under tVNS in normal subjects has been explored by several fMRI studies (Kraus et al., 2007, 2013; Dietrich et al., 2008; Frangos et al., 2015; Yakunina et al., 2017; Badran et al., 2018; Sclocco et al., 2019), allowing human subjects to avoid radiation hazards from imaging using such methods as SPECT and PET, although the cables connected to stimulation electrodes (when they are placed in a circular pattern and generate an electrical current) can burn the skin in the contact area. These studies used different stimulation parameters and demonstrated diverse results other than deactivation of the limbic system (the amygdala, hippocampus, and parahippocampal gyri) (Kraus et al., 2007, 2013; Frangos et al., 2015; Yakunina et al., 2017). Additionally, activation in the thalamus, cerebellum, insula, and frontal gyrus (Kraus et al., 2007, 2013; Dietrich et al., 2008; Frangos et al., 2015; Yakunina et al., 2017; Badran et al., 2018) has been reported frequently. Activation of the LC and NTS in the brainstem is also often considered robust evidence of vagal activation (Dietrich et al., 2008; Frangos et al., 2015; Yakunina et al., 2017). Figure 2 demonstrates activation of the LC and NTS following tVNS at the tragus and concha, but not the infero-posterial ear canal or earlobe (Yakunina et al., 2017). A recent ultrahigh-field 7T fMRI study explored the brainstem response to tVNS delivered during exhalation and found activation in the NTS, LC, and raphe nuclei (Sclocco et al., 2019). The authors suggested that exhalation-gated tVNS enhances NTS targeting.

A noteworthy difference between brain activation induced by tVNS and that by VNS is deactivation of the auditory cortices (superior and middle temporal gyri) following tVNS but not VNS (Kraus et al., 2007; Yakunina et al., 2017). Deactivation of these temporal cortices might reflect multisensory integration of the somatosensory and auditory systems. The medullary somatosensory nuclei receive sensory inputs from cranial and cervical nerves including the trigeminal, facial, glossopharyngeal, vagal, C1, and C2 nerves and then project to the dorsal cochlear nucleus in the auditory system (Young et al., 1995). This auditory–somatosensory connection seems to be involved more with ABVN than with the other branches of the vagus nerve. Electrical stimulation of the earlobe (innervated mostly by the cervical nerve) induces similar auditory cortex deactivation (Yakunina et al., 2017), possibly via the same multisensory integration. Therefore, the earlobe may not be a good sham location for tVNS studies. Earlobe stimulation has been used in cranial electrotherapy stimulation, which is FDA approved for the treatment of insomnia, depression, and anxiety and induces deactivation in several brain regions that overlap with those deactivated by tVNS (Yakunina et al., 2017).

## (t)VNS Effects in Patients With Tinnitus

### Neuroimaging Studies of tVNS in Tinnitus Patients

Lehtimaki et al. (2013) performed magnetoencephalography (MEG) while stimulating tinnitus patients with tVNS; a pure tone matched to the tinnitus frequency was presented continuously under both tVNS-on and tVNS-off conditions (Table 2). The amplitude of auditory N1m responses to the tone was reduced following the application of tVNS. This was a pilot study performed on only eight subjects; no control group was used. Another MEG study used the same stimulation setup but included a control group of normal hearing subjects who underwent sham tVNS at the earlobe (Hyvarinen et al., 2015). Tinnitus patients had higher beta- and gamma-band synchrony compared with the control group at baseline. tVNS at the tragus induced a reduction in beta and gamma synchrony in accordance with tinnitus severity. Sham tVNS had only a weak effect on the normalized spectrum at frontal alpha and beta and no effect on the measures of synchrony. The amount of gamma-band synchronization in the human auditory cortex was correlated with the subjective loudness of tinnitus (Van Der Loo et al., 2009). The authors concluded that tVNS was successful in modulating tinnitus-related beta- and gamma-band activity and thus could have potential in the treatment of tinnitus. The study was done on a small sample size (7–8 subjects).

**TABLE 2:** Summary of neuroimaging studies on (t)VNS in patients with tinnitus.

Study	Method	Stimulation	Study group	Control group	Application time	Stimulation parameters	Results	Safety/side effects
Lehtimaki et al. (2013)	MEG	tVNS (tragus)	tVNS-on, tVNS-off, + tone matched with Ftinn *n* = 8	–	One session tVNS-on tVNS-off; Duration NS	∼0.8 mA (above sensory threshold) 25 Hz	tVNS decreased the amplitude of the auditory N1m response.	Possible acute adverse effects were not monitored during MEG
Hyvarinen et al. (2015)	MEG	tVNS (tragus)	tVNS-on, tVNS-off, + tone matched with Ftinn *n* = 7	tVNS in normal subjects (earlobe), +1 kHz tone *n* = 8	One session 6 min active 6 min sham	∼ 0.5 mA (just above sensory threshold) 25 Hz 500 μs	tVNS modulates the synchrony of tone-evoked tinnitus-related brain activity, especially at the beta and gamma bands.	NS
Yakunina et al. (2018)	fMRI	tVNS (tragus, concha)	tVNS *n* = 36	tVNS in normal subjects (earlobe) *n* = 37	Two sessions per location 30 s on 30 s off 5 min per session	0.3–3.0 mA 0.71 ± 0.43 tragus, 0.80 ± 0.47 concha (just below pain threshold) 25 Hz 500 μs	tVNS deactivated multiple brain areas related to tinnitus generation and related distress. Both locations activated the vagal pathway in the brainstem.	No adverse effects

*EEG, electroencephalography; fMRI, functional magnetic resonance imaging; Ftinn, tinnitus frequency; MEG, magnetoencephalography; NS, not stated.*

**FIGURE 3:**
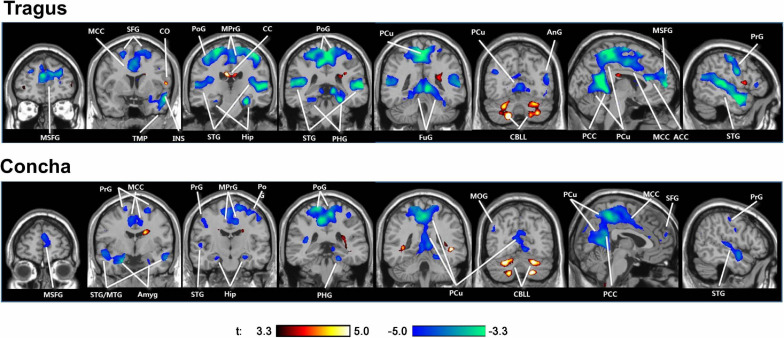
Activations (red) and deactivations (blue) induced by tVNS at the tragus and cymba conchae in tinnitus patients. tVNS resulted in deactivation of the auditory and limbic systems, as well as a number of other cortical areas. ACC/MCC/PCC, anterior/middle/posterior cingulate cortex; Amyg, amygdala; AnG, angular gyrus; CC, corpus callosum; CBLL, cerebellum; FuG, fusiform gyrus; Hip, hippocampus; LiG, lingual gyrus; MOG, middle orbital gyrus; MTG/STG, middle/superior temporal gyrus; PCu, precuneus; PoG/PrG, postcentral/precentral gyrus; SFG, superior frontal gyrus; TMP, temporal pole.

The only extant tVNS fMRI study in tinnitus patients found that tVNS deactivated the auditory and limbic areas (Figure 3; Yakunina et al., 2018). Numerous neuroimaging studies supported Jastreboff’s neurophysiological model, which suggests an abnormally strong connection between the auditory and limbic systems in tinnitus patients (Jastreboff, 1990; Chen et al., 2017). Various other non-auditory brain areas associated with tinnitus were also deactivated by tVNS, such as the cingulate cortex, precuneus, and frontal gyrus.

It should be noted that both controlled studies used the earlobe for sham stimulation which, as discussed above, may not be a good location for sham tVNS.

Together, these findings indicate that tVNS suppresses tinnitus-related brain networks, reduces auditory N1m responses, and reduces the level of beta- and gamma-band synchrony, which is elevated in individuals with tinnitus and is correlated with tinnitus loudness. Therefore, neuroimaging results suggest that tVNS affects brain activity in a way that potentially reduces the generation of tinnitus and tinnitus-related distress.

### Clinical Studies of (t)VNS Paired With Sound Stimuli in Tinnitus Patients

Since the 2011 study by Engineer et al. (2011) on VNS paired with tones in a rat model, numerous studies on direct and tVNS with and without paired sound stimuli in tinnitus patients have been published. Among them, seven studies explored tVNS paired with sound stimuli (Table 3), of which six explored the efficacy of paired tVNS for reducing tinnitus symptoms, and the remaining study explored the effects of paired VNS on voice and hearing. Five and two of the seven studies evaluated VNS and tVNS, respectively.

**TABLE 3:** Summary of the existing studies on (t)VNS paired with sound stimuli in patients with tinnitus.

Study	Stimulation	Study group	Control group	Application time	Stimulation parameters	Explored variables	Results	Safety/side effects
De Ridder et al. (2014a)	VNS	VNS + tones without Ftinn *n* = 10	–	20 days 2.5 h/day 2-month follow-up	0.8 mA 100 μs 30 Hz Every 30 s	THI MML	Average THI decreased by 11.78% (28.17 in the no-drug group). Clinically significant improvements in 4 of 10.	Post-surgery complications in two patients (redness; vocal cord hypomobility; infection)
De Ridder et al. (2015)	VNS	VNS + tones without Ftinn *n* = 1 (case report)	Tones *n* = 1 (same subject)	20 days 5 d/week 2.5 h/day	0.8 mA 100 μs 30 Hz Every 30 s	THI TRQ THQ TAQ MML	THI reduced by 48% TRQ reduced by 68% Control: both THI and TRQ increased.	No adverse effects
Tyler et al. (2017)	VNS	VNS + tones without Ftinn *n* = 16	VNS *n* = 14	6 weeks 7 d/week 2.5 h/day	0.8 mA 100 μs 30 Hz 0.5-s train delivered every 30 s	THI	Active group had significantly improved THI, while control group did not. THI improved in 50% of active group vs. 28% of controls. VNS + tones is more effective for subgroup of tinnitus patients that do not have hissing or blast-induced tinnitus.	Mild, well-tolerated adverse effects; similar profile to VNS in epilepsy
Vanneste et al. (2017)	VNS	VNS + tones without Ftinn *n* = 18	–	4–12 weeks 2.5 h/day	0.8 mA 100 μs 30 Hz 0.5-s train delivered every 30 s	EEG THI VAS loudness	Reduced gamma band activity in the left auditory cortex and reduced phase coherence between the auditory cortex and areas associated with tinnitus distress. Significant reduction of THI and VAS.	NS
Kochilas et al. (2020)	VNS	VNS + tones without Ftinn *n* = 7	VNS and tones separately	6 weeks 2.5 h/day	0.8 mA 100 μs 30 Hz 0.5-s train delivered every 30 s	Voice characteristics; PTA; MML; tinnitus pitch; monosyllabic word recognition	VNS + tones have no adverse impact on voice. Slight PTA threshold increases were observed at 2, 3, and 4 kHz.	Minimal or no adverse effects
Lehtimaki et al. (2013)	tVNS (tragus)	tVNS + TMNM *n* = 10	–	10 days 7 sessions, each session 45–60 min	∼0.8 mA (above sensory threshold) 25 Hz	WHO-5-point VAS THI Mini-TQ	Increased WHO well-being scores (56 → 76); THI, mean VAS loudness and annoyance, mini-TQ reduced significantly.	No adverse effects observed
Shim et al. (2015)	tVNS (concha)	tVNS + TMNM *n* = 30	–	10 sessions, each session 30 min, 1–4 days between sessions	1–10 mA (highest possible) 200 μs 25 Hz	GI index VAS loudness Tinnitus awareness score THI	GI score improved in 50% of patients. Mean loudness and awareness improved significantly THI improved, but not significantly (41.5 → 35.4).	No adverse effects reported

*Ftinn, tinnitus frequency; GI, global improvement; Mini-TQ, Mini Tinnitus Questionnaire; MML, Minimum masking level; NS, not stated; PTA, pure tone average; TAQ, Tinnitus Activities Questionnaire; THI, Tinnitus Handicap Inventory; THQ, Tinnitus Handicap Questionnaire; TMNM, tailor-made notched music; TRQ, Tinnitus Reaction Questionnaire; VAS, visual analog scale; WHO-5, World Health Organization-Five Well-Being Index.*

All five VNS studies paired VNS with pure tones that excluded the tinnitus frequency (De Ridder et al., 2014a, 2015; Tyler et al., 2017; Vanneste et al., 2017; Kochilas et al., 2020). The study durations ranged from 3 to 12 weeks. VNS parameters were identical in all studies. In the only randomized double-blind trial (Tyler et al., 2017), after 6 weeks of VNS paired with tones, the scores on the Tinnitus Handicap Inventory (THI) were significantly improved in the active group compared with controls who received VNS only. Improvement was seen in 50% of the participants in the paired VNS group compared with only 28% of controls. However, the sample size was small (16 patients in the VNS group). The other controlled study used partial data from a previous study exploring the effect of paired VNS on voice and showed that paired VNS does not have a negative effect on voice in tinnitus patients (Kochilas et al., 2020). One of the most common side effects of VNS is its possible effect on voice; it can reduce vocal cord motion on the implantation side with secondary supraglottic muscle tension, causing voice changes or hoarseness (Al Omari et al., 2017).

A case report of a patient with chronic tinnitus unresponsive to various previous therapies (De Ridder et al., 2015) showed improvement in tinnitus-related symptoms that lasted for 2 months after treatment with VNS paired with tones without tinnitus frequency.

Other VNS studies were uncontrolled and recruited too few subjects. Four of ten subjects exhibited clinically meaningful improvement in their tinnitus [THI and minimum masking level (MML)] (De Ridder et al., 2014a) that lasted more than 2 months after therapy. They claimed that the patients on medications that might interfere with VNS-released neuromodulators showed no improvement in their tinnitus. The same group retrospectively analyzed the results of EEG done before and immediately after 1–3 months of VNS paired with tones (Vanneste et al., 2017). The study group was a subset of patients from two previous VNS + tones trials (De Ridder et al., 2014b; Tyler et al., 2017). VNS–tone pairing reduced gamma-band activity in the left auditory cortex. The reduction in gamma-band activity was correlated with the degree of loudness reduction. Paired VNS also reduced the phase coherence between the auditory cortex and areas associated with tinnitus distress, including the cingulate cortex and the parahippocampus. The authors argued that these results support the hypothesis that VNS paired with sound stimuli could direct therapeutic neural plasticity.

Two uncontrolled clinical trials investigated the feasibility of tVNS paired with sound stimuli (TMNMT) for tinnitus treatment. Ten sessions of paired tVNS at the tragus in 10 subjects showed significant reductions in the mean THI and Mini Tinnitus Questionnaire (mini-TQ) scores, and subjective loudness and annoyance were also decreased by more than 20 points (Lehtimaki et al., 2013). The other study applied 10 sessions of paired tVNS at the concha in 30 subjects; the mean subjective loudness and tinnitus awareness score (but not THI) were significantly improved after treatment (Shim et al., 2015).

Overall, direct or tVNS paired with sound stimuli seems to produce a positive therapeutic effect on reducing tinnitus symptoms. However, small sample sizes, the absence of controls, and the lack of long-term follow-up seriously limit the reliability of these studies. Furthermore, given the wide heterogeneity of tinnitus pathophysiology and symptoms, well-organized systematic studies are needed to establish the effects of paired tVNS, particularly in different subgroups of tinnitus patients.

No adverse effects have been observed in tVNS studies, but VNS studies have often reported some adverse effects related to the invasiveness of the procedure (Table 3). VNS is reportedly a well-tolerated and relatively simple surgical procedure, but it is costly and carries the risk of side effects such as cough, hoarseness, voice alteration, and paresthesia (Ben-Menachem, 2001). Both direct and tVNS present cardiac-related risks, such as bradycardia and cardiac asystole (Ben-Menachem, 2001). Therefore, patients with cardiac disorders and implanted devices such as pacemakers should be screened out, and heart rate should be monitored during the stimulation periods.

### Clinical Studies on (t)VNS Alone in Tinnitus Patients

Several studies have investigated the effects of stand-alone tVNS in tinnitus patients (Table 4). The cardiac safety of long-term tVNS in tinnitus patients (Kreuzer et al., 2012) was demonstrated by ECG in subjects without cardiac disease. In another study, tVNS alone induced no clinically relevant improvements in tinnitus-related complaints even after 24 weeks of treatment (Kreuzer et al., 2014). Suk et al. (2018) applied four sessions of 4-min tVNS at each of three stimulation sites for 2 weeks. At 4 weeks after the end of treatment, tinnitus-related visual analog scale (VAS) scores were improved by at least 50% in 33–45% of the patients, and THI and Beck Depression Inventory scores also improved significantly. The authors reported no significant differences in tinnitus relief according to tVNS intensity; a stepwise increase in intensity up to the tolerable maximum level did not produce significant differences in the treatment outcome. However, in addition to the absence of a control group, the short treatment duration (4-min session at each location for 2 weeks) was different from other tVNS protocols, which performed treatment for several hours each day for several weeks (Table 3). Wichova et al. (2018) retrospectively evaluated epilepsy patients with tinnitus who received VNS as a treatment for their epilepsy. Phone inquiries regarding changes in the loudness of tinnitus (VAS) showed that 16 of 20 patients had at least one quieter moment. Furthermore, the difference between pre- and postoperative loudness was statistically significant. However, the results of that study were difficult to interpret reliably because of the retrospective nature, fact that the preoperative VAS was recorded postoperatively, inconsistent VNS settings among patients, absence of a control group, small sample size, and unknown treatment period, among other factors.

**TABLE 4:** Summary of the existing studies on (t)VNS alone (not paired with sound stimuli) in patients with tinnitus.

Study	Stimulation	Study purpose	Sample size	Application time	Stimulation parameters	Primary variable	Results	Safety/side effects
Kreuzer et al. (2014)	tVNS (site NS)	Feasibility, safety, efficacy for tinnitus treatment	*n* = 24 in phase 1, *n* = 26 in phase 2	Phase 1: 30 s on/180 s off 6 h/day 45.5 ± 21.0 days Phase 2: 30 s on/30 s off 4 h/day 24 weeks	0.1–10 mA (just above sensory threshold) 25 Hz	TQ THI TBF-12 BDI VAS ECG	No clinically relevant improvement of tinnitus complaints. tVNS considered safe in patients with no history of cardiac disease.	Twitching and pressure at electrode placement site. No significant changes in cognitive testing.
Suk et al. (2018)	tVNS (cavum, concha, tragus)	Clinical significance of tVNS intensity in tinnitus	*n* = 24	4 min per site sequentially; 4 sessions over 2 weeks	Intensity increased by 1 mA every 5 s until maximum without pain 30 Hz 200 μs	THI BDI VAS	VAS scores improved at least 50% in 33.3–62.5% of patients THI and BDI were reduced significantly. No effect of stimulus intensity on treatment outcome	No adverse effects
Wichova et al. (2018)	VNS	Effect on perception of tinnitus in epilepsy patients	*n* = 20	NS	NS	VAS loudness	Post-operative VAS loudness decreased significantly. At least a one-point decrease in VAS loudness in 80% of patients	Transient post-operative hoarseness in one patient
Kreuzer et al. (2012)	tVNS (site NS)	Cardiac safety in tinnitus	*n* = 24	30 s on/180 s off 5.15 ± 1.80 h/day 24.0 ± 19.3 days	3.2 ± 2.5 (0.1–10) mA (just above sensory threshold) 25 Hz	ECG	tVNS is feasible in tinnitus patients without signs of long-term worsening of tinnitus complaints. In subjects with no known pre-existing cardiac pathology no arrhythmic effects of tVNS were seen.	Two adverse cardiac events (one severe) registered, but considered very unlikely to be tVNS-induced
Ylikoski et al. (2017)	tVNS (site NS)	Acute effects on autonomic nervous system imbalance	*n* = 97	One session 15–60 min	0.1–130 mA (above sensory threshold) 25 Hz 250 μs	HRV	tVNS can induce a shift from sympathetic preponderance toward parasympathetic predominance	No major morbidity, no cardiac or circulatory effects during active tVNS
Ylikoski et al. (2020)	tVNS (tragus)	Clinical features, psychophysiological characteristics, and HRV	tVNS *n* = 171 tVNS + TRT *n* = 78	One session 15–60 min for HRV assessment/60–90 min/day 5 d/week 1 year	0.3–3.0 mA (above sensory threshold) 25 Hz 250 μs	HRV TQ	tVNS improved parasympathetic function, most efficiently in patients with a low starting HRV level. tVNS + TRT alleviated tinnitus stress and handicap in 76% of patients	No cardiac adverse effects in 250 tVNS patients

*None of the studies had a control group. BDI, Beck Depression Inventory; ECG, electroencephalography; Ftinn, tinnitus frequency; HRV, heart rate variability; NS, not stated; TBF-12, Tinnitus Impairment Questionnaire; THI, Tinnitus Handicap Inventory; TRT, tinnitus retraining therapy; TQ, Tinnitus Questionnaire; VAS, visual analog scale.*

Ylikoski et al. (2017, 2020) evaluated acute tVNS effects on the autonomic nervous system in tinnitus patients by measuring heart rate variability before and after a tVNS session. They concluded that tVNS induces a shift in autonomic nervous system functioning from sympathetic preponderance toward parasympathetic predominance, thus reducing the stress-related imbalance in the autonomic nervous system. A consistent improvement in heart rate variability, which is considered a useful marker of mental stress, was observed in 90% of the patients (Ylikoski et al., 2017, 2020). The authors argued that tVNS can be a helpful therapeutic tool in reducing tinnitus-related mental stress. Their second study reported significant decreases in loudness and tinnitus-related annoyance in 78 tVNS-treated patients; however, all patients simultaneously received tinnitus retraining therapy (TRT), a form of instructive counseling combined with sound therapy using white noise. Thus, the tVNS effect could not be evaluated separately from the counseling effect of the tinnitus retraining therapy.

Engineer’s method for tinnitus treatment using VNS, based on the tonotopic model of tinnitus, involves paired sound stimuli; it is the sound stimuli that initiates rearrangement of the auditory cortex to eliminate the origin of tinnitus percept (Engineer et al., 2011). VNS enforces this process through action of several neuromodulators, thus promoting neural plasticity. Therefore, (t)VNS alone is not expected to be effective as tinnitus therapy following the original “targeted plasticity” method. Nevertheless, a number of studies attempted to demonstrate that tVNS alone without any paired stimuli can be successful in relieving tinnitus symptoms. The main reasoning behind these attempts is that tVNS modulates the auditory and limbic areas, as shown by neuroimaging studies in normal subjects and tinnitus patients. Additional arguments include VNS positive effect on habituation, its antidepressant mode of action and influence on vegetative nervous system, and clinical data on electrical auricular stimulation that might have involved unintentional vagal stimulation (Kreuzer et al., 2014). tVNS also reduces imbalance of the autonomic nervous system related to tinnitus-induced stress (Ylikoski et al., 2020).

Nevertheless, no strong or clear evidence that tVNS alone reduces tinnitus-related symptoms is currently available. However, this does not mean that tVNS has no potential as a tinnitus-relief therapy but rather points to the need for more reliable studies to provide robust evidence of its ability to relieve tinnitus.

## Discussion

VNS paired with sound stimuli as tinnitus treatment was first proposed by Engineer et al. (2011). The study was performed on rats; tinnitus was induced by exposing rats to loud octave-band noise centered on a certain frequency. Inability of noise-exposed rats to detect silent gaps in narrow-band noise centered on the assumed tinnitus frequency was taken as a behavioral correlate of tinnitus; the gap impairment was eliminated after rats were continuously treated with VSN paired with tones excluding the frequency of tinnitus. To the best of our knowledge, all existing studies replicating these results are from the same group of authors (Shetake et al., 2012; Engineer et al., 2015, 2017; Buell et al., 2019; Adcock et al., 2020). No replication of this work by an independent research team exists. This is the first and most glaring gap in existing literature on the subject; the entire field of VNS paired with sound stimuli as a therapy for tinnitus is based on work by a single research team. The first and most urgent need is to replicate and verify these results.

Furthermore, the method is based on the tonotopic model of tinnitus which claims that auditory cortical reorganization is a primary cause for tinnitus. This model has multiple flaws and has been strongly criticized. Among arguments against it is the fact that tinnitus can develop without preceding extensive hearing loss which is generally required for cortical reorganization; no human study demonstrated the evidence for cortical reorganization in tinnitus patients; tinnitus often occurs instantaneously within a few seconds following noise exposure which is not enough time to generate tonotopical plasticity in the cortex (see Knipper et al., 2013 for a review). Additionally, overwhelming majority of studies exploring the relationship between the tinnitus pitch and audiometric edge frequency found no connection between the two variables (see Yakunina and Nam, 2021 for a review). Hence, while we cannot exclude the possibility that cortical map reorganization may be the reason for developing tinnitus in some individuals, it is by far nor necessary neither always existing condition for tinnitus.

Despite the fact that VNS is normally a well-tolerated and relatively simple surgical procedure, it nevertheless is invasive. tVNS is a non-invasive and thus cheaper, safer and easier-to-implement alternative. A new invasive experimental therapy should generally not be used in human subjects until there is a strong effectiveness evidence base. Therefore, (paired) tVNS rather than VNS for tinnitus therapy should receive primary focus in future research, particularly given its own challenges such as finding a proper site for active and sham stimulation as discussed above. Before VNS is considered for tinnitus treatment, the effectiveness of tVNS for tinnitus should be firmly established.

As of now, there is no evidence showing effectiveness of either treatment in relieving tinnitus as all existing studies are of poor quality, which is confirmed by a recent systematic review (Stegeman et al., 2021). Future studies should follow the Consolidated Standards of Reporting Trials (CONSORT) recommendations, which provide guidelines for designing and reporting randomized control trials that include double blinding and a suitable sample size (Schulz et al., 2010). The calculation of the sample size is one of the most important steps in designing a randomized controlled trial; it majorly influences the statistical reliability of the findings. And yet, this step is most frequently omitted in existing clinical studies. At the same time, individual results should not be neglected and are encouraged to be reported, since there are many different subtypes of tinnitus, and the reaction to treatment can vary widely depending on individual tinnitus and psychological characteristics (Tyler et al., 2007, 2008). Primary and secondary variables should be carefully chosen based on the existing considerations for the design of tinnitus clinical trials (Tyler et al., 2006, 2007).

## Conclusion

Direct VNS or tVNS paired with sound stimuli excluding the individual’s tinnitus frequency appears to have potential as a treatment method for alleviating tinnitus symptoms; however, no reliable study exists on this topic as yet. All existing studies have major flaws such as the absence of a control group, small sample size, and lack of randomization and blinding. Similarly, there is no reliable evidence to date showing that (t)VNS alone without paired sound stimuli is effective for tinnitus treatment. Higher-quality research is needed for both paired and unpaired tVNS to compensate for the flaws of existing studies and address the gaps in the current knowledge on the subject, such as proper stimulation parameters, longer follow-up periods, and the most responsive tinnitus populations.

## Author Contributions

NY wrote the manuscript. E-CN curated and supervised the study, and edited the manuscript. Both authors contributed to the article and approved the submitted version.

## Conflict of Interest

The authors declare that the research was conducted in the absence of any commercial or financial relationships that could be construed as a potential conflict of interest.
